# Process evaluation of a randomised controlled trial of PBS-based staff training for challenging behaviour in adults with intellectual disability

**DOI:** 10.1371/journal.pone.0221507

**Published:** 2019-08-22

**Authors:** Alessandro Bosco, Laura Paulauskaite, Ian Hall, Jason Crabtree, Sujata Soni, Asit Biswas, Vivien Cooper, Michaela Poppe, Michael King, Andre Strydom, Michael J. Crawford, Angela Hassiotis

**Affiliations:** 1 Institute of Mental Health, University of Nottingham, Nottingham, United Kingdom; 2 Division of Psychiatry, University College London, London, United Kingdom; 3 Community Learning Disability Service, Mile End Hospital, East London NHS Foundation Trust, London, United Kingdom; 4 Community Learning Disability Service, Camden and Islington NHS Foundation Trust, London, United Kingdom; 5 Leicestershire Partnership NHS Trust & University of Leicester, Leicester, United Kingdom; 6 Challenging Behaviour Foundation, the Old Courthouse, Chatham, Kent, United Kingdom; 7 Department of Forensic and Neurodevelopmental Sciences, Institute of Psychiatry, Psychology and Neuroscience, King's College London, United Kingdom; 8 South London and the Maudsley NHS Foundation Trust, London, United Kingdom; 9 Department of Medicine, Imperial College London, London, United Kingdom; University of Auckland, NEW ZEALAND

## Abstract

**Background:**

Positive Behaviour Support (PBS) for challenging behaviour is a complex intervention. Process evaluation is pivotal in fully understanding the mechanisms and contextual factors that impact on participant outcomes.

**Aims:**

To conduct a process evaluation of a national clinical trial investigating the impact of PBS-based staff training on the level of challenging behaviour in adults with intellectual disability.

**Method:**

The Medical Research Council guidance for process evaluation of complex interventions was followed. Semi-structured interviews with 62 stakeholders from the intervention arm (service users, family and paid carers, service managers, staff who delivered the intervention and PBS trainers), quantitative data from the study database and an external evaluation of the quality of the PBS plans were used.

**Results:**

Twenty-one health staff volunteered to be trained in delivering PBS. Available log data from 17 therapists revealed that they worked with 63 participants a median of 11.50 hours (IQR 8–32). Only 33 out of 108 reports had included all elements of the intervention. Another 47 reports had some elements of the intervention. All PBS plans were rated weak, indicating insufficient quality to impact challenging behaviour. Stakeholders reported an appreciation of PBS and its potential to impact quality of care and engagement with the participant. However, they also identified important challenges including managing PBS-related caseloads, paid carer turnover and service commitment to the delivery of PBS.

**Conclusions:**

PBS-based staff training was well received, but therapists found it difficult to undertake all the elements of the intervention in routine care. Implementing a workforce training strategy is important to better define the active components of PBS, and resource implications if the intervention is no better than usual care.

## Introduction

Between 10–15% of people with intellectual disability (ID; a lifelong condition manifesting during early development) engage in behaviours that challenge [1, 2]. Individuals with ID have significantly increased vulnerability to biological, psychological and environmental stressors compared to the general population [3]. A reduced ability to cope with these stressors makes individuals with ID more likely to engage in challenging behaviour [3, 4]. Challenging behaviours are remitting and relapsing, and maybe of intensity, duration and frequency that not only negatively impact the life of individuals with ID but also of those who support them [5, 6].

Positive Behaviour Support (PBS) is a therapeutic framework which involves a number of evidence-based interventions appropriate to the needs of the individual. It is a first line approach in supporting individuals with ID who display challenging behaviour in the UK and elsewhere [7–10]. It consists of multiple interacting elements (including identification of the function of challenging behaviour) and multiple agents that are required for its implementation (such as direct care staff and professionals). An important constituent element is Applied Behaviour Analysis [11]. An overarching objective of PBS is to enhance quality of life by understanding the function of challenging behaviours leading to personalised interventions for family and paid carers about how to prevent and/or reduce the occurrence of such behaviours [11].

Support for the efficacy of staff-training in PBS primarily comes from design N = 1 experimental or uncontrolled studies, with there being a limited number of RCTs that examine its clinical and cost effectiveness [12, 13]. One pilot RCT found that PBS is effective in reducing challenging behaviour when delivered by specialist behavioural teams compared to standard multidisciplinary community intellectual disability teams [14]. However, this type of care is often unavailable [15]. Therefore, in order to increase access to Positive Behaviour Support, training carers and frontline staff in its delivery is an essential and necessary approach. There is preliminary evidence from a systematic review, indicating a significant reduction in challenging behaviour among individuals with ID in the presence of PBS-based staff training interventions [16].

For the purposes of this paper however, we describe PBS as a complex intervention, considering it as one therapeutic entity, which consists of different elements. Complex interventions occur in environments whereby multiple interacting factors could impact on the delivery of the intervention and on its effectiveness (e.g. multiple agents involved in the delivery of care) [17, 18, 19]. Therefore, the evaluation of the processes involved when delivering PBS is pivotal in better understanding which characteristics of the intervention are associated with high fidelity and sustainable delivery and which may act as barriers [20]. The Medical Research Council (MRC) framework for process evaluation of complex interventions recommends investigating the intervention context, implementation and mechanisms in order to provide additional context especially where findings show that interventions have not been effective above and beyond usual care [21].

Although deemed important, to date no process evaluation has been conducted for this type of intervention to highlight which mechanisms may be associated with patient outcomes. Partly, this is due to the process evaluation being introduced relatively recently as a component of clinical trials and partly due to the small numbers of randomised controlled trials in the field of intellectual disabilities and interventions in particular. It is also to be acknowledged, that process evaluation may help better design future RCTs for the management of challenging behaviour in adults with ID [22]. The clinical trial started a year after the Winterbourne scandal exposed the poor care and abuse perpetrated on adults with ID who displayed challenging behaviour in an inpatient facility in England [23]. During the three years of the study, the care of people with ID underwent gradual changes driven by strong family and patient advocacy and the Government response to address long term systemic failures in the care of people with ID [24–27]. Services who participated in this trial were located in England covering semi-rural, rural and urban areas. A median of 500 service users were registered with each of the services which took part in the study and a median of 23 full-time health care staff were employed by those services [28]. The study found that there was no significant difference in the reduction of challenging behaviour between the intervention and treatment as usual groups over 12 months [28].

The aim of the process evaluation, employing the MRC framework for process evaluation of complex interventions, was to investigate the implementation (i.e. the training and delivery of PBS) through analysis of (i) fidelity (i.e. the extent to which PBS following staff training was implemented and delivered as planned), (ii) dose (i.e. how much of PBS was delivered), (iii) reach (i.e. the number of participants receiving PBS), and (iv) adaptations (i.e. the extent to which PBS was tailored to participants). A further aim was to explore the contextual factors that might influence the outcome of the intervention and identify the different mechanisms of the PBS-based staff training that may lead to the present outcomes. For clarity, in this paper we used the terms ‘PBS trial’ to differentiate between the clinical effectiveness which has been reported elsewhere [28], and ‘process evaluation’, reported here which has evaluated the implementation, the mechanisms of impact and context of the intervention delivery and implementation.

## Materials and methods

### PBS trial

Twenty-three community ID services (total N = 246 people with ID and challenging behaviour) in England, UK, were randomly allocated to either the intervention (N = 11) condition or treatment as usual (TAU; N = 12) condition. Twenty-six volunteer health staff in the intervention condition were trained in PBS approaches (see main PBS trial paper for further methodological detail [28]. The staff who trained in PBS approaches, hence referred to as therapists, came from a number of professional backgrounds (11 nurses, 5 occupational therapists, 5 psychiatrists, 2 speech and language therapists, 1 clinical psychologist, 1 assistant psychologist and 1 social worker). The PBS trial received ethical approval by the National Research Ethics Service Committee London-Harrow (reference 12/LO/1378). All participants who had capacity provided written consent before participation in the trial; family or nominated consultees gave assent for those participants without capacity.

Participants in the intervention group received PBS as well as treatment as usual (TAU). The latter was defined as routine care that is available within the community ID services including multidisciplinary input from a variety of health and social care professionals, e.g. psychiatrists, psychologists, nursing.

### Process evaluation–Implementation study

Stakeholders taking part in the qualitative interviews were derived from the main trial [28]. This included participants with mild to moderate ID and verbal ability, family and paid carers, service managers and therapists. We also used data regarding reach and dose of therapy collected as part of the clinical effectiveness study.

In order to illustrate how the PBS-based training might work and produce the anticipated outcomes, we developed a logic model adapted from the Kirkpatrick’s Four-Level Training Evaluation Model (1959) [29]. The model was also used as a reference framework to gather participants’ views about the quality and effectiveness of the intervention that they received and this has been reported elsewhere [30].

The logic model levels are:

1. Inputs- this domain describes what has to be in place in order for the PBS-based staff training and associated interventions to take place.
2. Processes- this domain describes the delivery of the training and the implementation of the intervention.
3. Actions- the domain establishes the key elements of the PBS-based training intervention which need to be implemented in order to produce the study outcomes.
4. Results- the domain outlines the anticipated outcomes of the intervention.

The outcomes of the process evaluation were assessed using qualitative semi-structured interviews with stakeholders who took part in the study. In addition, quantitative data from the study database was used as well as an external independent evaluation of the quality of PBS plans. The PBS plans (a central output of the PBS-based training) are developed from what has been learnt from the functional assessment or analysis of the behaviour. They detail the approaches that family or paid carers should take to adapt the environment and support participants to develop alternative skills and behaviours to replace challenging behaviour.

### Independent quality assessment

An independent assessor (Board Certified Behaviour Analyst and Associate Fellow of the British Psychological Society with 35 years of experience) assessed the quality of PBS plans developed by the therapists in the study. The Behaviour Intervention Plan Quality Evaluation Scoring Guide II (BIP-QE II) was applied to examine whether the 12 key domains of behaviour plan were present in the plans (Table 1) [31]. Each of the 12 key domains was given a score of 0 (no information), 1 (some information) or 2 (all necessary information). Higher scores on the BIP-QE II indicate an increased likelihood that a behaviour plan and the interventions or approaches contained within it would be implemented with fidelity [31].

**Table 1:** Behaviour intervention plan quality evaluation.

Elements of evaluation	
A. Challenging behaviour is identified in observable and measurable terms B. Predictors/ triggers of challenging behaviour C. Analysis of what supports challenging behaviour D. Environmental changes that eliminates problem behaviour E. Predictors related to function of challenging behaviour F. Function related to replacement behaviours G. Teaching strategies H. Reinforcement for positive behaviour I. Reactive strategies J. Goals and objectives K. Team coordination L. Communication	
**Score out of max 24**	
Fewer than 12 points = **Weak Plan**	This plan may effect some change in challenging behaviour but the written plan is weak and should be rewritten.
13–16 points = **Underdeveloped Plan**	This plan may effect some change in challenging behaviour but would require a number of alterations.
17–21 points = **Good Plan**	This plan is likely to effect a change in challenging behaviour.
22–24 points = **Superior Plan**	This plan is likely to effect a change in challenging behaviour and embodies best practice.

### Mechanisms of impact and contextual factors study

Data for the mechanisms of impact and contextual factors study derived from individual semi-structured interviews with a purposive sample of 13 participants with intellectual disability who had sufficient verbal ability, 11 family carers, 10 paid carers, 12 service managers, 12 therapists and a focus group with the 4 PBS trainers. These interviews were conducted to assess the context in which the intervention was delivered and stakeholders’ views of both the intervention and the training components. We obtained informed consent from all participants; easy read versions were used for the participants with mild to moderate intellectual disability. Researchers and the service user reference group (Camden Advocacy Project SURGE) developed topic guides triangulating available literature and the aims and objectives of the study which were piloted and changes were made as needed. The interviews were audio recorded and transcribed verbatim by the lead author (AB) who was not directly involved in either the delivery of the intervention nor the data collection. All text from study interviews were managed using the qualitative research software NVivo 10 [32].

The interview transcripts were coded and analysed according to the inductive method of thematic analysis described by Braun and Clark (2006) during which, themes and subthemes are data driven and emerge from the text and are not predefined by researchers [33]. By doing so, the analytic process involved the reading and re-reading of data for each broad theme or ‘topic’ related to the experience of participants of the PBS intervention, without being influenced by any previous research on behavioural interventions in ID population. Topics were given the status of ‘theme’ when it emerged more than twice from the transcript. Any topics which appeared less than twice from the text but were still relevant for the present work were discussed prior to a decision being made whether to include them in the analysis. Themes were processed at the ‘latent level’ with each theme being analysed beyond the pure descriptive and sematic level of narration [33]. Once the themes and sub-themes were generated, the researcher created a codebook for co-raters to test inter-rater reliability measured using Cohen’s Kappa coefficient and based on the parameters proposed by Landis and Koch, 1977. Co-raters were a Clinical Psychologist, three Consultants in the Psychiatry of ID and a family carer of a person with ID. Inter-rater reliability was in the range of 0.8–1, indicating an almost perfect agreement [34]. The co-raters undertook three rounds of codebook revisions to determine themes and subthemes. The service user advisory group was consulted on five different occasions in this process to cover the preparation of questions for the qualitative interviews; the exploration of the preliminary findings of the interviews with stakeholders and to gather final comment on the interpretation of the findings.

## Results

Our findings have been structured in a linear way from a general overview to a more specific reporting of the results for each component of the process evaluation. For this reason, we first included a summary of findings ensuing from our process evaluation and we then reported separately on the implementation and the mechanisms of impact and contextual factors.

Therapists were unable to work with 7 participants who, however, provided outcome data to the study. Five of these participants already had PBS plans developed by external agencies as accommodation providers disclosed at the point of contact that they were employing a PBS practitioner. Additionally, two family carers declined to work with the therapists.

### Implementation (resource and training)

Manual-assisted face-to-face training was delivered by expert PBS trainers in three two-day workshops over a period of 15 weeks. Out of the 26 therapists who volunteered to receive the training, 5 therapists dropped out at the beginning of the training (3 due to illness, 1 left their position and 1 for unknown reason) and were not further involved in the study. Twenty-one started the intervention work, but 7 therapists did not remain in post until the end of the study (1 due to maternity leave, 4 left their positions or were seconded to another position, 1 took sabbatical leave and 1 due to illness). None of these 7 therapists were replaced as it was not possible to carry out more training events.

Prior to inclusion in the PBS study each service agreed to reduce therapists’ routine caseload by around 20% (1 day a week) to allow for the addition of PBS work with a maximum of 7 participants. However, although each therapist recorded the time spent on delivering the intervention to his/her allocated number of participants with ID, no information was recorded on whether and to what extent their service related caseload was reduced sufficiently to allow them to work more intensively with the trial participants. It is clear from the interviews with the therapists, that the agreed reduction in caseload did not happen in the majority of cases. That is, the therapists were expected to work as usual and in addition carry out the study tasks.

Post-training mentoring for the time that a participant was in the study was available to the therapists who were responsible for utilising this support as required, but also were prompted by regular emails or phone calls by the study administrator who also liaised with the trainer mentors. The trainers provided feedback on plans, and problem solving regarding the delivery of PBS. Several meetings took place with service managers and therapists to promote buy-in and ongoing support for the therapists both before and after the study started. The research team offered assistance with collection of study related paperwork. Clinical supervision and case management remained with the clinical teams.

### Fidelity, dose and reach

For each participant the therapists were expected to complete a Brief Behavioural Assessment Tool (BBAT); conduct observations to inform the functional analysis/assessment, to formulate a PBS Plan; complete the Goodness of Fit in discussion with the carers to ensure implementation and support problem solving. Finally, therapists completed the Fidelity Checklist (designed to assess how many of the anticipated elements of the PBS intervention they had completed).

Out of the 108 possible datasets that should have contained all of the above elements, 24 were fully completed, 47 were partially completed and for 37 participants there were no therapist datasets. The reasons for the missing or incomplete data are presented in Table 2. Among the reasons for missing intervention data, was a mild or absent level of challenging behaviour in participants with ID (n = 16) at the time that the therapist approached the participant and his/her carers to begin the intervention. The 33 “Goodness to Fit” forms which rated the effectiveness of the PBS plan received from 9 clusters, indicated that the family/paid carers found the plans helpful and confirmed that they were able to implement them in the long term (“in next 12 months”). We did not, however, perform any observations of the plan implementation by carers.

**Table 2:** Number of full and partial datasets and reasons for missing data.

**Completed data sets and reasons for missing date**	(N, %*)
**Full data set**	24 (22.2%)
**Partial or missing data set**	84 (77.7%)
**Reasons for missing data**	(N, %*)
Workload too large	23 (21.2%)
Challenging Behaviour (none or mild at point of contact)	16 (14.8%)
Organisational issues impacting on study (external PBS providers, guidelines already in place, participants under the care of another team/professional, managers unwilling to assist with workload management)	14 (12.9%)
Staff leaving	12 (11.1%)
Participant/family carer did not want to engage with the therapists	7 (6.4%)
Referral to other team/service	2 (1.8%)
Information unavailable	10 (9.2%)
**Total**	**84 (77.7)**

* Percentage of datasets out of total datasets (N = 108).

Each therapist also completed a Log of engagement detailing the tasks and time spent in relation to the delivery of PBS. At the time of receiving training, the therapists were advised by the PBS trainers that an estimated 12.5 hours would be required per case. Available Log of Engagement data for 17 therapists revealed that they spent a median of 11.50 hours (IQR 8–32) per participant relating to the intervention broken down into the following tasks (median given): 0.5 hours for direct contact or observations of a participant; 3.5 hours for contact with family, paid carers and/or service at the assessment stage; 2.3 hours for PBS plan writing and study related paperwork; 1.5 hours for contact with family, paid carers and/or service at the intervention stage; 2 hours for further indirect work not accounted in other areas (e.g. travel).

### Independent quality assessment

All complete PBS plans and a selection of incomplete work (N = 61) were sent to the external assessor to assess the quality of the work involved in the delivery of PBS using the Behaviour Intervention Plan Quality Evaluation Scoring Guide II (BIP-QE II). The assessment revealed that the 33 completed plans (representing 30.6% out of a total of 108 datasets) were all rated as low quality (weak plans) and unlikely to have an impact on challenging behaviour. Three main reasons were identified: 1) lack of evidence that functional analyses had been conducted; 2) lack of evidence of specific interventions (such as skills teaching or differential reinforcement) being carried out; or 3) information on factors that maintained the challenging behaviour not being recorded.

### Mechanisms of impact and contextual factors study: Stakeholder experience of PBS-based staff training

Six main themes emerged from the interviews with stakeholders.

#### 1. Impact on organisation

Most therapists and managers reported a strong motivation to implement a PBS approach to challenging behaviour:

*‘…I already have a couple of cases of people presenting with quite severe challenging behaviour and other lighter*, *less time-intensive interventions hadn’t proved helpful*. *And absolutely*, *it was helpful’*. (Clinical psychologist 0102, female)

Therapists reported that the intervention enabled the service to co-ordinate different approaches delivered by a multidisciplinary team:

*‘So*, *for example*, *we might have speech and language therapy in the course and that speech and language therapy can be integrated into the PBS approach*, *for what the PBS approach does is that hopefully all the people involved in that person’s care are aware of the goals they’re trying to achieve’*. (Community ID nurse 0811, female)

However, some therapists also reported that the intervention was similar to the approach they already used in their practice:

*‘The PBS and what we do are so the same that the only real difference was the tool we used and then how we wrote it up in the first person’*. (Community ID nurse 0915, female)

#### 2. Engagement during the study

This theme describes a range of experiences the stakeholders had in regard to the support and communication with the therapists during the implementation study. Positive support was reported by family, paid carers and participants with ID:

*‘The quality of the help I would say*, *it was excellent*. *V*. *(therapist) is a professional…and as I see her intervention has helped in the sense that*, *yeah has improved A*.*‘s (participant with ID) life for sure and ours’*. (Paid carer 040510, female)

The family carers also experienced a positive response to the therapist input:

*‘You know*, *it gets just a bit…you feel worn out when he gets a bit fed up*. *So if you have got somebody coming in to encourage you*, *it cheers you up again’*. (Family carer 111904, mother)

However, stakeholders also mentioned instances where they felt disappointed when they did not receive adequate support.

*‘I do not know why we did not receive the intervention*. *Maybe*, *they felt we were managing his behaviour*, *but how to manage his behaviour*, *then that is the crucial question’*. (Family carer 040504, female)

#### 3. Implementation

The PBS-based training was experienced positively by the majority of therapists:

*‘I think the training we have been having was excellent… really clear*, *great examples*. *People took us through the things step by step… it was really practical in terms of what we have done’*. (Speech and language therapist 0405, female)

Certain aspects of the work with the therapists was seen as helpful; the use of daily time tables was singled out as enabling paid or family carers to engage participants in different activities and improve the quality of support. Therapists reported that the PBS plan enhanced the understanding of the participant’s needs.

*‘For example… before now*, *he could not*, *maybe…let’s say make a cup of tea*, *but with the PBS we have broken it down into steps… and now when you talk to him and say ‘bring milk’ he knows to go to the fridge*, *open the fridge and he knows how to recognise milk’*. (Paid carer 050610, female)

Participants with ID recognised when a family member or paid carer was trying to help when s/he was in distress:

*‘Yeah*, *because my mom knows how to make me calm’*. (Participant 112005, female)

*‘He (paid carer) talks to me so ‘what is your problem*?*’ Let me know what your problem is so that I can help you’*. (Participant 060704, male)

Managers reported that time management was consistently a challenge:

*‘I think the issue was competing priorities*, *so when you have got day-to-day operational work and research work where the priority goes*, *I mean people quite often go to the operational end and I think start trying to do the research work around that and that time has been difficult’*. (Manager 0405, male)

The therapists mentioned that by the time they were ready to begin work with a participant, either the individual’s behaviour had improved, or paid carer turnover affected the delivery of the intervention and data collection.

*‘We found that many of them (participants) when we came to visit them*, *we found that actually there wasn’t any current challenging behaviour*. *So*, *possibly there had been some issues in the past*, *but at the current time they were very settled*, *so then it was hard to find a clinical rationale to prioritise them above somebody else who was presenting a new or some kind of really challenging…’*. (Clinical psychologist 0102, female)

*‘Staff (paid carers) turnover had an impact on being able to implement the PBS plan or even develop it if there is a change in people and community staff’*. (Speech and language therapist 0405, female)

#### 4. Managing difficult situations

Family and paid carers said that as a result of having been involved in the trial, they had either learnt different strategies to manage challenging behaviour or reflected about the individual’s behaviour:

*‘I think if you do not re-direct somebody away from what they are anxious about*, *then they are going to continue to get anxious about that…the trick is there*, *so if you re-direct them and move away from it until the trick has gone and then re-direct them and focus them on something else*, *but it has to be a positive focus*.*’* (Paid carer 091306, female)

*‘We did a lot of talking about S*.*’s everyday life and everything he did he likes to do and what he likes and what he dislikes which obviously the therapist was putting together to do the PBS plan’*. (Family carer 111904, mother)

Therapists emphasised the importance of trying to understand the behaviour, and to consider a variety of reasons why an individual may display challenging behaviour:

*‘Although she has the same behaviour*, *it is actually as if the function is different*, *so it is usually when she cannot do something and thinks she finds it hard or she finds it hard being asked to do something that she does not want to do’*. (Occupational therapist 0506, female)

#### 5. Research impact

Taking part in the trial was associated with challenging therapists’ and managers’ views on how to manage behaviour effectively:

*‘I have been able to actually reduce some of the medications and depot medications*, *so the frequency and severity of those behaviours have been significantly reduced’*. (Consultant Psychiatrist 1018, female)

*‘It makes a difference to the staff in the way (they) think about things…so people who have done the training they do*, *they approach things slightly differently*, *and think things through quite differently’*. (Manager 0102, female)

Both service managers and therapists also expressed concern with the resources needed for the delivery of PBS, for example completing several assessment forms and other paperwork, hence the impact this had on the time they could spend on routine clinical care:

*‘My experience of that was slightly frustrating for the clinician*, *because of the amount of time the work around the research took*. *So my experience was to try to negotiate with people within the service and to try to have free time for the individual’*. (Manager 0102, female)

*‘It would be interesting to look at the figures actually with an estimate of how many hours we would be…working with each client and I think it was roughly under-represented because… somebody has to do the work for us*, *there were three people in my little cohort and we were all shocked about the amount of work that we were asked to undertake’*. (Occupational therapist 1120, female)

#### 6. Understanding of the concepts of PBS

Most therapists said that they had developed a good understanding of PBS and how to conduct a behavioural assessment, which was deemed a fundamental starting point in the understanding of causes behind challenging behaviour.

*‘It is a capacious and open approach and it’s just that kind of beginning part*, *making sure that you’ve done a really good assessment and you have a good understanding*, *a kind of starting point for why and what the function of the behaviour is’*. (Clinical Psychologist 0102, female)

#### PBS Trainers overview

The PBS trainers (N = 4), external to the study, had many years of experience in delivering PBS training to a variety of health and social care professionals and had developed the manual for the intervention. They were each allocated 4–5 therapists and maintained contact with them via emails and telephone calls. The trainers also took part in monthly teleconferences which ran for 12 months (2014–2015).

Absence of service support to therapists being involved in the study was seen as the main challenge which also had an impact on the development of the PBS plans.

*‘If you were the participant who is being trained by us but you are working in a service context that isn’t supportive of what we are trying to do then… your power to affect change is hugely reduced’*. (Trainer 4, male)

*‘There is practice leadership issue*. *She [therapist] was trying to get things implemented in a staff team and there was no manager or the manager was not ever there…we provided a lot of training …but ultimately is the management with all staff that will implement PBS plan without our support’*. (Trainer 1, male)

Further, the trainers raised the issue of paid carers leaving their jobs and the impact that had on the delivery of the intervention.

*‘The issue was a very high staff turnover in one site in particular so she [therapist] talked to one staff one day who the week later no longer worked and she did some training with some staff*, *you know*, *never seen again really*, *and these concerns were so serious that escalated locally’*. (Trainer 1, male)

The trainers argued that the challenging behaviour displayed by the study participants may have been less severe and therefore, not the major focus for concern or in need for intervention and that the diverse professional background of the therapists may have contributed to the challenges in delivering certain aspects of the intervention, e.g. functional assessments/analysis of the behaviour.

*‘There is no behaviour at present and it is such a shame the way some teams are supposed to have PBS and not allocated suitable people for the study’*. (Trainer 1, male)

*‘One of the issues we all thought was that people with very different roles attended the training…not many had some prior knowledge of PBS and struggled to see the role of using the BBAT to achieve functional analysis and struggled to fit the PBS plan within their role’*. (Trainer 3, male)

A summary of findings from the process evaluation is presented in Table 3.

**Table 3:** Summary of methods and findings from the process evaluation of the PBS-based staff training based on the MRC guidance [21].

Outcome measure	Definition	Data source	Main findings
**The intervention context**	External barriers or facilitators that might influence the delivery of the intervention.	Discussion with sites prior taking part in the trial	*Facilitators*: offer of PBS training; buy-in for staff who volunteered to receive the training. *Barriers*: managers supporting the therapists in terms of study-related caseload, time constraints and paid carer turnover rates.
**Implementation process**	Resources and support essential for the intervention to be delivered.	Study database	26 therapists committed to attend the training: 5 dropped out during the training; 7 left during the study but were not replaced. Support regarding the delivery of PBS was offered from the research team for the duration of the study (individual mentoring, monthly peer support teleconferences, site visits) and mentoring support from PBS trainers.
**Fidelity**	Extent to which the intervention was delivered as intended.	Study database; quality evaluation of the PBS plans	Out of 108 datasets: 33 complete and 47 incomplete. For 28 datasets no data received. 61 PBS related elements assessed: 33 scored below 12 (weak quality) and 28/61 had no evidence of PBS formulation. Plans lacked functional analyses, skills teaching or range of high quality interventions.
**Dose**	Amount of the intervention delivered.	Study database	17 therapists spent a median of 11.5 hours (IQR 8–32) on each participant. *Assessment stage*: a median of 3.50 hours on observations; 3.50 hours for contact with family, paid carers and/or service; 2.15 hours for further indirect work. *Delivery of the intervention*: a median of 2.29 hours for PBS plan writing and study related paperwork; 1.50 hours for contact with family, paid carers and/or service.
**Reach**	Number of participants that had received the intervention.	Study database	Out of a possible 108 participants, therapists were able to work with 80 (74%).
**Adaptations**	Changes made to the intervention to reach a better fit with the context.	Semi-structured interviews with stakeholders	No specific adaptations were reported when delivering PBS; however, therapists did not undertake work with participants who lived in accommodation that employed own therapists outside of the study
**Mechanisms of impact**	Stakeholders’ perceived benefits and challenges of the intervention.	Semi-structured interviews with stakeholders	Therapists appreciated the training. Family, paid carers and service managers reported increased knowledge of PBS and of participants’ with ID needs. Participants with ID had some understanding of strategies that helped them to become less agitated or upset. However, paid carer turnover, clinical caseloads and perceived lack of support from the services were seen as substantial obstacles in delivering the training and intervention in routine care.

## Discussion

This work is the first process evaluation of a PBS-based staff training for adults with ID who display challenging behaviour. Results from our trial revealed that the PBS-based staff training did not reduce challenging behaviour for people with ID compared to treatment as usual [28]. The recruitment of participants was completed in 2015 and therefore, several enhancements to the delivery of PBS that have since been implemented were not available at the time. The landscape of care in services for people with ID and challenging behaviour has changed considerably as a result of policies and driven by family carer demand for better care and care standards. Therefore, whilst currently a system-based approach to training in PBS is being rolled out, this was not the focus of our trial. In this instance the process evaluation was carried out simultaneously with a pragmatic randomised controlled trial that was contracted to deliver pre-specified outcomes. We can, however, propose a view as to why the intervention may not have been implemented as desired due to a number of dynamic and interacting factors including lack of a systems-based approach and lack of periodic monitoring. There were 11 community ID teams in the intervention arm and we did not identify a single team that appeared to perform better than others in intervention delivery. This is robust evidence that the issues of implementation were (and possibly still are) widespread and must be resolved urgently.

The PBS based training and the approach to the treatment of challenging behaviour was well received by most stakeholders. Therapists, managers and carers reported that they understood the function of behaviour better having taken part in the study. However, several aspects of PBS related work were frequently omitted by therapists resulting in low fidelity of the intervention; further, the therapists found routine care pressures insurmountable in addition to carrying out the research tasks. The PBS plans often lacked the detail needed to deliver the intervention comprehensively and therefore, PBS was not delivered as initially intended. Further, treatment as usual may have contained elements that were very similar to PBS as recommended in best practice. PBS plans, where available, were valued by family and paid carers as a helpful tool that enabled the understanding of the participants’ needs.

We found that following mentor contact, site visits by the researchers and monitoring teleconferences, there was an increase in study related activity, for example completion of study paperwork or tasks.

Service issues remained a challenge throughout the study. Prior to commencing the study, all community ID services that were eligible and interested in taking part were visited by the chief investigator to discuss the requirements of the study. Five co-investigators were members of individual community ID services and promoted the study although there was no formal implementation phase undertaken. Allocation to the intervention arm appeared to create difficulties for the services, which had to accommodate a different approach to their delivery of routine care including releasing staff to attend 2–3 days of training in London on three occasions. Therapists in almost all the intervention sites were unable to provide the estimated commitment of one day per week to engage in PBS work related to the trial. However, the study achieved good participant recruitment (as per the sample size calculation) and retention (90%) with certain elements of implementation achieved (e.g. training, support and engagement with 74% of the participants). The retention rate was better than is usually seen in reports of evaluations of psychosocial interventions either in ID or other populations [35].

Additional challenges were identified through the process evaluation. Due to the issues described above, many therapists experienced time constraints in delivering the intervention and reported receiving little support from their service managers. The trainers indicated that some of the therapists did not utilise the mentoring available for skill enhancement and embedding of knowledge. The training delivered in the study was longer than most courses offer (approximately 1–3 days) and included face to face contact and case preparation. Therapists may have benefited from receiving also a training component around leadership and management for implementing interventions within their service. Such a component may have helped the therapists in gaining the skills to effectively monitor the progress of implementation of PBS within their team (e.g. through group team discussion) by correcting actions, and by the use of contingent rewards mechanisms provided as necessary. In addition, on-site support, outside of training, was not provided to teams although, monthly teleconferences were conducted as described earlier. Although trainers were available when needed for mentoring and problem solving, the therapists found difficult to navigate their time in the study in addition to other demands as in some cases they were not as well supported as they could have been by their managers. Several therapists, who were all working in the services, made plans to leave the service for sabbaticals or other posts and that impacted the number of participants that were seen for the study; in effect, that meant that when a therapist left, his or her study cases were not being taken over by the remaining therapist. This is likely to be an issue in actual practice whereby trained therapists leave their posts, consequently, making the delivery of PBS unachievable or less than optimal. The PBS Academy in the UK has now published standards for providers and community services in an attempt to improve and standardise PBS delivery within services and by practitioners (http://pbsacademy.org.uk/pbs-standards/).

Paid carer turnover was a contributing factor in delays in undertaking assessments and implementing PBS plans. Our study gives the strongest indication yet that providers must be aware that time and context (i.e. high staff turnover, low staff levels, high caseloads etc.) may have consequences for the delivery of PBS.

However, those difficulties are not unique. For example, the Walk Well study reported that organisational factors, e.g. low support, staff turnover and the burden of study related paperwork possibly led to inadequate intervention delivery [36]. Another multicomponent health intervention for people with ID found that the effectiveness of the intervention depended on staffing levels, work climate and overall service leadership [37].

As this is the first large scale independent RCT of PBS-based staff training, there is no comparable literature to examine process outcomes, especially in controlled conditions [38]. We calculated that only half an hour was given to direct observations, but we are unable to compare it to other studies or even routine care as such information is lacking. It is also possible that the approaches outlined in the PBS trial therapist training was similar to the multidisciplinary approach to the treatment of challenging behaviour already available in several services.

In terms of limitations of our process evaluation the reliability and validity of the BIP-QE II tool used to assess the quality of the PBS plans has only been documented in educational settings with a young population without intellectual disability [39]. However, participants in this trial were adults with ID living in a variety of community settings (i.e. supported living, residential care). Thus, it is unclear whether this tool was able to capture the quality of the PBS plans reliably in this study. Additionally, the PBS training was not based on the BIP-QE II tool and perhaps some of the areas that are included in the tool were not covered during the training.

Further, the researchers did not carry out direct observations of PBS plan implementation by carers and therefore, even when carers appeared to be committed to carry out the plan in the longer term, this cannot be verified. A formal supervision, provided through video feedback for example, may have increased the understanding of carers over patterns of their behaviours or that of the person they were caring for, that required improvement to reach adequate levels of implementation of the PBS plan.

Finally, in exploring the stakeholder experience of the PBS trial we were only able to interview individuals with mild ID with sufficient verbal ability; therefore, experiences of individuals with more severe ID may have been missed and not captured even by interviews of paid or family carers.

### Lessons learnt and future research

The process evaluation provides useful insights into the barriers that might have influenced the delivery of PBS as it is clear that despite the fact that substantial support was provided to therapists the intervention was delivered with low fidelity. In the light of our process evaluation findings, we argue that there is urgent need to tackle the barriers to the implementation of PBS in order to optimise its delivery, which as discussed in the paper were:

- Service workloads/pressures
- Duration of intervention delivery and competing clinical responsibilities
- Paid carer turnover rates
- Insufficient managerial support
- Skill development of therapists in formulating PBS plans
- Role and timing of direct observation of the participant
- Mentoring and supervisory support on site

It is also important to define PBS core components (what can PBS change or influence?) and the impact of other mental health comorbidities which may require specific interventions within its framework in order to improve patient outcomes. For example, a significant proportion of participants in the trial were assessed with such comorbidities; therefore, timely diagnosis and treatment of those conditions is essential to avoid further impairment in adaptive functioning.

Other important issues for consideration include the high paid carer turnover, the resources required for training for the delivery of the intervention if staff undertake this work in addition to core duties, and the supervisory arrangements. Without a comprehensive approach to further investigating the mechanisms of the onset, course and prognosis of challenging behaviour [40–42] training in PBS may only address the needs of a proportion of all people with ID and of their families impacted by challenging behaviour.

It is recognised that often interventions that rely on therapist training and complex systems of delivery can be difficult to sustain long term [43]. This may help explain why many complex interventions that are found to have efficacy in single site phase II trials fail to demonstrate clinical effectiveness when tested at scale [43]. Hence, training staff members in PBS, or train a sole agent to deliver the intervention across services could be associated with long term implementation. This is an important point and although outside the scope of the present study it would need to be considered as a question to be answered in future research.

Future studies examining implementation of PBS should focus on long term sustainability in its delivery, particularly in social care facilities or in family homes. In addition, researchers may need to consider adaptive trial designs that allow for more flexibility in the conduct of a clinical trial [44–46]. Therefore, every effort must be made to ensure that any such schemes translate into high fidelity implementation in routine care. This concern is further borne out by the fact that whilst the stakeholders thought highly of the PBS training and that participants and provider services found the engagement with the therapists and the PBS strategies acceptable and useful, nevertheless, those elements did not improve fidelity. Current work in developing guidance on how to support effectively people with complex needs may also be an important contributor to the questions as to how best to implement such multicomponent frameworks such as PBS which depend on an indirect approach to working with individuals. Practice leadership, which is thought to be essential in good provider support, should be promoted as an enabling factor in ensuring services that are fit for purpose [47]. Whilst there is an important argument to be made about the need to censure high fidelity and improve training standards, there remains to be decided as to whether a less formal PBS approach as is likely to be taking place currently in community ID services, confers enough gains at least for the majority of adults who display challenging behaviour [48].

## References

[1] Bowring DL, Totsika V, Hastings RP, Toogood S, Griffith GM. Challenging behaviours in adults with an intellectual disability: A total population study and exploration of risk indices. Br J Clin Psychol. 2017;56(1):16–32. 10.1111/bjc.12118 27878840

[2] American Psychiatric Association. Diagnostic and Statistical Manual of Mental Disorders 5th ed Arlington, VA: American Psychiatric Publishing; 2013.

[3] Allen D. Positive behavioural support as a service system for people with challenging behaviour. Psychiatry. Elsevier Ltd; 2009;8(10):408–12.

[4] Grey IM, Hastings RP. Evidence-based practices in intellectual disability and behaviour disorders. Curr Opin Psychiatry [Internet]. 2005;18(5):469–75. Available from: http://content.wkhealth.com/linkback/openurl?sid=WKPTLP:landingpage&an=00001504-200509000-00003. 10.1097/01.yco.0000179482.54767.cf 16639103

[5] Emerson E, Kiernan C, Alborz A, Reeves D, Mason H, Swarbrick R, et al The prevalence of challenging behaviors: A total population study. Res Dev Disabil. 2001;22(1):77–93. 11263632 10.1016/s0891-4222(00)00061-5

[6] Green VA, O’Reilly M, Itchon J, Sigafoos J. Persistence of early emerging aberrant behavior in children with developmental disabilities. Res Dev Disabil. 2005;26(1):47–55. 10.1016/j.ridd.2004.07.003 15590237

[7] Royal College of Psychiatrists. Challenging Behaviour: A Unified Approach London: Psychological Society and Royal College of Speech and Language Therapists 2007.

[8] NICE Guidance. Challenging behaviour and learning disabilities: prevention and interventions for people with learning disabilities whose behaviour challenges. 2015. Available from: https://www.nice.org.uk/guidance/ng11/resources/challenging-behaviour-and-learning-disabilities-prevention-and-interventions-for-people-with-learning-disabilities-whose-behaviour-challenges-1837266392005. 26180881

[9] Disability Services Commission, Government of Western Australia. Positive Behaviour Support Information for Disability Sector Organisations. 2012. Available from: http://www.disability.wa.gov.au/Global/Publications/For%20disability%20service%20providers/Guidelines%20and%20policies/Behaviour%20Support/Positive%20Behaviour%20Support%20Information%20Sheet%20for%20Disability%20Sector%20Organisations.pdf.

[10] Ravensberg H. IDEA 2004: Final Regulations and the Reauthorized Functional Behavioral Assessment. 2004 Available from: http://www.pbis.org/comon/cms/files/pbisresources/SSRN_d1151394.pdf.

[11] Gore NJ, McGill P, Toogood S, Allen D, Hughes JC, Baker P, et al. Definition and scope for positive behavioural support. International Journal of Positive Behavioural Support. 2013;3(2):14–23. Available from: http://www.ingentaconnect.com/content/bild/ijpbs/2013/00000003/00000002/art00003.

[12] Didden R, Korzilius H, Oorsouw W van, Sturmey P. Behavioral Treatment of Challenging Behaviors in Individuals With Mild Mental Retardation: Meta-Analysis of Single-Subject Research. Am J ment [Internet]. 2006;111(4):290–8. Available from: http://www.aaiddjournals.org/doi/abs/10.1352/0895-8017(2006)111[290:BTOCBI]2.0.CO;2. 10.1352/0895-8017(2006)111[290:BTOCBI]2.0.CO;2 16792430

[13] Lavigna GW, Willis TJ. The efficacy of positive behavioural support with the most challenging behaviour: The evidence and its implications. J Intellect Dev Disabil. 2012;37(3):185–95. 10.3109/13668250.2012.696597 22774760

[14] Hassiotis A, Robotham D, Canagasabey A, Romeo R, Langridge D, Blizard R, et al Randomized, single-blind, controlled trial of a specialist behavior therapy team for challenging behavior in adults with intellectual disabilities. Am J Psychiatry. 2009;166(11):1278–85. 10.1176/appi.ajp.2009.08111747 19687128

[15] McClean B, Dench C, Grey I, Shanahan S, Fitzsimons E, Hendler J, et al Person Focused Training: a model for delivering positive behaviour supports to people with challenging behaviours. J Intellect Disabil Res. 2005; 49: 340–352. 10.1111/j.1365-2788.2005.00669.x 15817051

[16] MacDonald A, McGill P. Outcomes of Staff Training in Positive Behaviour Support: A Systematic Review. J Dev Phys Disabil. 2013;25(1):17–33.

[17] Hieneman M. Positive Behavior Support for Individuals with Behavior Challenges. Behav Anal Pract [Internet]. 2015;8(1):101–8. Available from: http://link.springer.com/10.1007/s40617-015-0051-6. 10.1007/s40617-015-0051-6 27703893 PMC5048254

[18] Craig P, Dieppe P, Macintyre S, Michie S. Developing and evaluating complex interventions: the new Medical Research Council guidance. 2008;1655(September):1–6. 10.1136/bmj.a1655 PMC2769032 18824488

[19] Bonell C, Oakley A, Hargreaves J, Strange V, Rees R. Research methodology: Assessment of generalisability in trials of health interventions, suggested framework and systematic review. BMJ. 2006;333:346–9. 10.1136/bmj.333.7563.346 16902217 PMC1539056

[20] Hawe P, Shiell A, Riley T. Complex interventions: how “out of control” can a randomised controlled trial be? BMJ. 2004;328: 1561–63. 10.1136/bmj.328.7455.1561 15217878 PMC437159

[21] Moore GF, Audrey S, Barker M, Bond L, Bonell C, Hardeman W, et al Process evaluation of complex interventions: Medical Research Council guidance. Bmj [Internet]. 2015;350(mar19 6):h1258–h1258. Available from: http://www.bmj.com/cgi/doi/10.1136/bmj.h1258 25791983 10.1136/bmj.h1258 PMC4366184

[22] Hassiotis A, Strydom A, Crawford M, Hall I, Omar R, Vickerstaff V, et al Clinical and cost effectiveness of staff training in Positive Behaviour Support (PBS) for treating challenging behaviour in adults with intellectual disability: A cluster randomised controlled trial. BMC Psychiatry. 2014;14(1):1–10. 10.1186/s12888-014-0219-6 PMC4149205 25927187

[23] Department of Health. Transforming Care: A National response to Winterbourne View Hospital: Department of Health Review Final Report. 2012. Available from: https://www.gov.uk/government/uploads/system/uploads/attachment_data/file/213215/final-report.pdf.

[24] Department of Health. Winterbourne View Review: Concordat: A Programme of Action. 2012. Available from: https://www.networks.nhs.uk/nhs-networks/south-transforming-care/documents/winterbourne-view-concordat/view.

[25] NHS England. Building the right support: National Service Model. 2015. Available from: https://www.england.nhs.uk/learning-disabilities/natplan/.

[26] Royal College of Psychiatrist. People with learning disability and mental health, behavioural or forensic problems: the role of in-patient services. 2013. Available from: https://www.rcpsych.ac.uk/pdf/FR%20ID%2003%20for%20website.pdf.

[27] NHS Protect. Meeting Needs and Reducing Distress–Guidance on the prevention and management of clinically related challenging behaviour in NHS Settings. 2013. Available from: http://www.nhsemployers.org/news/2013/12/guidance-on-challenging-behaviour-published-by-nhs-protect.

[28] Hassiotis A, Poppe M, Strydom A, Vickerstaff V, Hall IS, Crabtree J, et al Clinical outcomes of staff training in positive behaviour support to reduce challenging behaviour in adults with intellectual disability: cluster randomised controlled trial. The British Journal of Psychiatry. Cambridge University Press; 2018;212(3):161–8. 10.1192/bjp.2017.34 29436314

[29] Kirkpatrick DL. Techniques for evaluating training programs. Journal of American Society of Training Directors. 1959;13:21–26.

[30] Hassiotis A, Poppe M, Strydom A, Vickerstaff V, Hall I, Crabtree J, et al Positive behaviour support training for staff for treating challenging behaviour in people with intellectual disabilities: a cluster RCT. Health Technol Assess. 2018;22(15). 10.3310/hta22150 29596045 PMC5900418

[31] Browning-Wright D, Mayer GR, Saren D. Behaviour Intervention Plan Quality Evaluation Scoring Guide II. The BIP Desk Reference. 2013;15: 3–39.

[32] NVivo qualitative data analysis Software; QSR International Pty Ltd. Version 10. 2012.

[33] Braun V, Clarke V. Using thematic analysis in psychology Using thematic analysis in psychology. Qual Res Psychol [Internet]. 2006;3(2):77–101. Available from: http://www.tandfonline.com/action/journalInformation?journalCode=uqrp20%5Cn 10.1191/1478088706qp063oa.

[34] Landis JR, Koch GG. The Measurement of Observer Agreement for Categorical Data Published by: International Biometric Society Stable URL: http://www.jstor.org/stable/2529310. 2016;33(1):159–74. 843571

[35] Abshire M, Dinglas VD, Cajita MIA, Eakin MN, Needham DM, Himmelfarb CD. Participant retention practices in longitudinal clinical research studies with high retention rates. BMC Medical Research Methodology. 2017;17(1):1–10. 10.1186/s12874-016-0277-1 28219336 PMC5319074

[36] Matthews L, Mitchell F, Stalker K, McConnachie A, Murray H, Melling C, et al Process evaluation of the Walk Well study: A cluster-randomised controlled trial of a community based walking programme for adults with intellectual disabilities. BMC Public Health [Internet]. BMC Public Health; 2016;16(1):1–11. Available from: 10.1186/s12889-016-3179-6. 27387203 PMC4936049

[37] Sundblom E, Bergström H, Ellinder LS. Understanding the Implementation Process of a Multi-Component Health Promotion Intervention for Adults with Intellectual Disabilities in Sweden. J Appl Res Intellect Disabil. 2015;28(4):296–306. 10.1111/jar.12139 25624120

[38] Parsons MB, Rollyson JH, Carolina N, Reid DH. Evidence-Based Staff Training: A Guide for Practitioners. Behavior Analysis in Practice 2012;5(2):2–11. 10.1007/BF03391819 23730462 PMC3592486

[39] Browning Wright D, Mayer GR, Cook CR, Crews SD, Kraemer B, Gale B. A Preliminary Study on the Effects of Training using Behavior Support Plan Quality Evaluation Guide (BSP-QE) to Improve Positive Behavioral Support Plans. Educ Treat Child. 2007;30(3):89–106.

[40] Melville CA, Johnson PCD, Smiley E, Simpson N, Purves D, McConnachie A, et al Problem behaviours and symptom dimensions of psychiatric disorders in adults with intellectual disabilities: An exploratory and confirmatory factor analysis. Res Dev Disabil [Internet]. Elsevier Ltd; 2016;55:1–13. Available from: 10.1016/j.ridd.2016.03.007 27018744

[41] Thygesen JH, Wolfe K, McQuillin A, Viñas-Jornet M, Baena N, Brison N, et al Neurodevelopmental risk copy number variants in adults with intellectual disabilities and comorbid psychiatric disorders. The British Journal of Psychiatry. Cambridge University Press; 2018;212(5):287–94. 10.1192/bjp.2017.65 29693535 PMC7083594

[42] Wolfe K, Stueber K, McQuillin A, Jichi F, Patch C, Flinter F, et al Genetic testing in intellectual disability psychiatry: Opinions and practices of UK child and intellectual disability psychiatrists. J Appl Res Intellect Disabil. 2018;31(2):273–84. 10.1111/jar.12391 28833975 PMC5836913

[43] Crawford MJ, Barnicot K, Patterson S, Gold C. Negative results in phase III trials of complex interventions: Cause for concern or just good science? British Journal of Psychiatry. Cambridge University Press; 2016;209(1):6–8. 10.1192/bjp.bp.115.179747 27369475

[44] Mahajan R, Gupta K. Adaptive design clinical trials: Methodology, challenges and prospect. Indian Journal of Pharmacology. 2010;42(4):201–207. 10.4103/0253-7613.68417 20927243 PMC2941608

[45] Korn EL, Freidlin B. Adaptive Clinical Trials: Advantages and Disadvantages of Various Adaptive Design Elements. J Natl Cancer Inst. 2017;109(6):1–6. 10.1093/jnci/djx013 PMC6279284 28376148

[46] Almirall D, Nahum-Shani I, Sherwood NE, Murphy SA. Introduction to SMART designs for the development of adaptive interventions: with application to weight loss research. Transl Behav Med. 2014;4(3):260–74. 10.1007/s13142-014-0265-0 25264466 PMC4167891

[47] Mansell J, Hughes H, McGill P. Maintaining local residential placements In Emerson E., McGill P., & Mansell J.(Eds.), Severe learning disabilities and challenging behaviours: Designing high-quality services (pp. 261–281). London, UK: Chapman and Hall; 1994.

[48] Dieterich M, Irving CB, Khokhar MA, Park B, Marshall M (2017). Intensive Case Management for severe mental illness. Cochrane Database of Systematic Reviews 2017, Issue 1. Art. No.: CD007906. 10.1002/14651858.CD007906.pub3 28067944 PMC6472672

